# Sanger sequencing in exonic regions of *STK11* gene uncovers a novel de-novo germline mutation (c.962_963delCC) associated with Peutz-Jeghers syndrome and elevated cancer risk: case report of a Chinese patient

**DOI:** 10.1186/s12881-017-0471-y

**Published:** 2017-11-15

**Authors:** Zi-Ye Zhao, Yu-Liang Jiang, Bai-Rong Li, Fu Yang, Jing Li, Xiao-Wei Jin, Shou-Bin Ning, Shu-Han Sun

**Affiliations:** 1 Department of Medical Genetics, Naval Medical University, 800 Xiangyin Rd, Shanghai, 200433 China; 2 Hebei North University, 11 South Zuanshi Rd., Zhangjiakou, Hebei Province 075061 China; 3 Department of Gastroenterology, Airforce General Hospital of PLA, 30 Fucheng Rd, Beijing, 100142 China

**Keywords:** Peutz-Jeghers syndrome, *STK11* gene, De-novo mutation, Hamartoma, Polyposis

## Abstract

**Background:**

Peutz-Jeghers syndrome (PJS) is caused by mutations in the tumor suppressor gene, *STK11*, and is characterized by gastrointestinal hamartomas, melanin spots on the lips and the extremities, and an increased risk of developing cancer.

**Case presentation:**

We reported an isolated PJS patient who died of colon cancer, whose blood sample was collected together with all the available family members’. The entire coding region of the *STK11* gene was amplified by PCR and analyzed by Sanger sequencing, through which, a novel mutation, c.962_963delCC in exon 8 was identified in this patient. This mutation causes a frameshift mutation and a premature termination at codon 358. Protein structure prediction by Swiss-Model indicated a dramatic change and partial loss of the C-terminal domain. We did not observe this mutation in both parents of the proband. Therefore, it is considered a novel de-novo mutation. Furthermore, the mutation was not found in 50 unrelated healthy people.

**Conclusions:**

The novel mutation we reported here had not been recorded in databases or literature, and the patient who possessed it suffered from PJS and colon cancer. So our results enlarge the spectrum of *STK11* variants in PJS patients. This mutation is most likely responsible for development of the PJS phenotype, especially the cancer occurrence.

**Electronic supplementary material:**

The online version of this article (10.1186/s12881-017-0471-y) contains supplementary material, which is available to authorized users.

## Background

Peutz-Jeghers Syndrome (PJS, OMIM 175200) is an autosomal dominant disorder characterized by gastrointestinal (GI) hamartomatous polyps, mucocutaneous pigmentation, and an increased risk for the development of GI and various extra-GI malignancies [1]. It is a rare condition with a low incidence of 1/50,0009 to 1/200,000 [2]. The elevated cancer risk in PJS patients has been observed in several cohort studies, and it is estimated to be 9–18 times higher than the general population [3, 4].

Since cloned by Jenne et al. and Hemminki et al. respectively in 1997, serine-threonine kinase 11 (*STK11/LKB1*, OMIM 602216) on chromosome 19p13.3 have been considered to be the pathogenic gene of PJS [5, 6]. The gene contains 10 exons, within which exon 1–9 encode a 433-amino-acid-residue protein, a tumor suppressor. Mutation detection rates of 10%–94% have been achieved at different centers, depending on the screening method, with considerable uncharacterized genetic heterogeneity remaining in this syndrome [7, 8]. Most mutations are frameshift or nonsense changes, which result in an abnormal truncated protein and the consequent loss of kinase activity, and they can be detected in PJS patients with or without family histories [9]. Different cancer risks due to different *STK11* mutations have been recognized through hundreds of cases reported previously, however the *STK11* mutation spectrum and genotype-phenotype correlation are still poorly understood.

Here, we report a novel mutation of *STK11* in a PJS patient without a family history, which is associated with definite cancer risk.

## Case presentation

### Clinical information

**Fig. 1:**
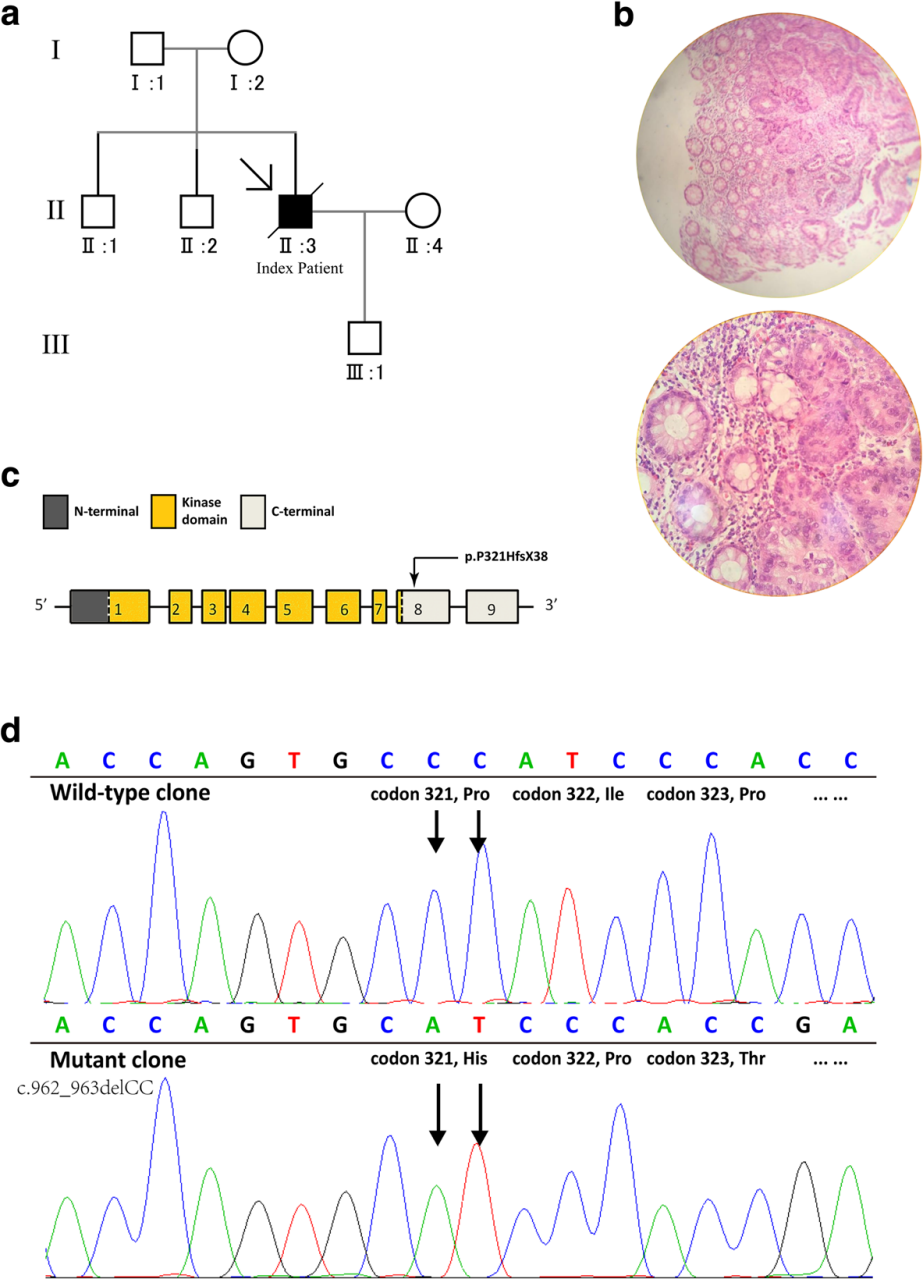
Genogram, pathology and electropherogram of the index patient. **a** The genogram showed an isolated PJS patient. Roman numerals indicate generations and Arabic numbers indicate individuals. Squares = males, circles = females. Affected individuals are denoted by solid symbols and unaffected individuals are denoted by open symbols. The index patient is indicated by an arrow. **b** Representative hematoxylin-eosin-stained tissue slices of the GI polyp biopsy specimens showed carcinoma infiltration. Up, ×100 magnification; low, ×400 magnification. **c** The structure of *STK11* gene. This novel mutation is within exon 8. **d** Sanger sequencing with the help of T vector assay revealed a heterozygous frameshift mutation, c.962_963delCC

A 46-year-old man from North China was diagnosed clinically with PJS at the age of 36 years old. The characteristic pigmentation on his lips and fingertips appeared at the age of 6. Diagnosis of PJS was determined after his partial ileal resection because of intestinal obstruction, and multiple hamartomatous polyps were confirmed in his postoperative pathological specimens. However, the patient had no relations to be affected, and his parents, two elder brothers and a 20-year-old only son had no PJS-related complaints (Fig. 1a and Additional file 1: Figure S1). The definitive diagnosis was made because this patient fulfilled two of the three clinical diagnosis criteria of PJS, which included the presence of characteristic mucocutaneous pigmentation, the presence of hamartomatous polyps in the GI tract, and a family history of PJS [10].

The patient came to our department at the age of 46, and complained abdominal distension and aggravating after meals. Multiple duodenal and colonic polyps with diameters as long as 2 cm were found by gastroscopy and colonoscopy. Sadly, pathologic findings of several resected colonic polyps revealed adenocarcinoma infiltration within hamartomas (Fig. 1b), and CT scan discovered small amount of ascites. After 2 month rest, the patient came back for further treatment, but the surgeons denied the feasibility of radical surgical treatment because of mass ascites, which indicated extensive abdominal metastasis. Another 2 months later, the patient passed away because of advanced colon cancer.

### Mutation analysis

Genomic DNA of peripheral blood leucocytes was extracted routinely by Isolation Kit (DP318, Tiangen Biotech, Beijing, China) according to the manufacturer’s instructions. All nine coding exons of the *STK11* gene were amplified by the use of primers listed in Table 1. PCRs of *STK11* exons were performed in a 50-ul reaction which contained 0.4 uM of each primer, 50 ng genomic DNA, and 25ul 2 × premix Ex Taq DNA polymerase (RR030A, Takara Bio Inc., Dalian, China). The PCRs were performed under the following conditions: denaturation at 95 °C for 4 min, followed by 35 thermal cycles, each composed of 95 °C for 30 s, 58 °C for 30 s, and 72 °C for 45 s.

**Table 1:** Primers for exon-specific sequencing of *STK11* gene

Exon	Forward primer (5′-3′)	Reverse primer (5′-3′)
1	CCGTTGGCACCCGTGACCTA	ACCATCAGCACCGTGACTGG
2	GGGCGGATCACAAGGTCA	AGGAGACGGGAAGAGGAGC
3	TGTGCCCAGAGCAAGAGC	GCAGAAGAATGGCGTGAACC
4 & 5	AGGAGACGGGAAGAGGAGC	TGAACCACCATCTGCCGTAT
6	TGACTGACCACGCCTTTCTT	TGAGGGACCTGGCAAACC
7	CAGGGTCTGTCAGGGTTGTCC	CCGTCCGCTGCTCTGTCTT
8	ACTGCTTCTGGGCGTTTGC	AGGTGGGCTGGAGGCTTT
9	GGTTCTGTGCTGGCATTTCG	GGCTCTGACGCTGGTGGAT
10a	TGCCCAGGCTGACCTCTTC	CGATGGCGTTTCTCGTGTTTT
10b	GGATTTGAGCTGTGGCTGTGAG	AACACCGTGACTGCCGACCT

The index patient and available family members (relatives I:1, I:2, II:1, II:2, II:4 and III:1) underwent *STK11* germline mutation testing to confirm cosegregation of the mutation with the disease. For frameshift mutation, T vector assay (CV15, Aidlab Inc., Beijing, China) was used to identify each haplotype by constructing monoclonal cells. In order to rule out polymorphisms and to confirm the pathogenic effects of variations, 100 chromosomes from 50 unrelated ethnicity-matched healthy individuals were also screened for the presence of the mutation. They were screenees from our department, and the age range was from 18 to 91 with an average age of 50.7.

The PCR products were gel- and column-purified and directly sequenced. The purified PCR fragments were then sequenced using BigDye Terminator (Applied Biosystems, Foster City, CA, USA) on an ABI Prism 3100 genetic analyzer (Applied Biosystems, Foster City, CA, USA) by Majorbio Co. Ltd.(Shanghai, China). The results were used to performance sequence alignment according to *STK11* gene sequence (NP_000446.1 and NM_000455.4 in GRCh38.p7).

Sanger sequencing revealed a heterozygous germline frameshift mutation in exon 8. Further, we performed T vector assay and identified the exact haplotype, which is a two-nucleotide deletion (c.962_963delCC) and has not been reported in literatures or recorded in mutation databases (Fig. 1c and d).

The mutation was only found in the index patient, but not in his healthy relatives and 50 unrelated healthy individuals. Since the mutation clearly co-segregates with the disease phenotype in the family, and the mutation is predicted to affect the key structure of STK11 protein, we conclude that this mutation is disease-specific and not a polymorphic variant of the *STK11* gene.

### Structure prediction of the mutant protein and analysis of evolutionary conservation of amino acid residues

The homology modeling programs, Swiss-Model (http://swissmodel.expasy.org), was used to develop an appropriate model to mimic the effects of the mutated region [11]. Evolutionary conservation of amino acid residues altered was analyzed by comparing across different species (https://www.ncbi.nlm.nih.gov/protein/STK11).

**Fig. 2:**
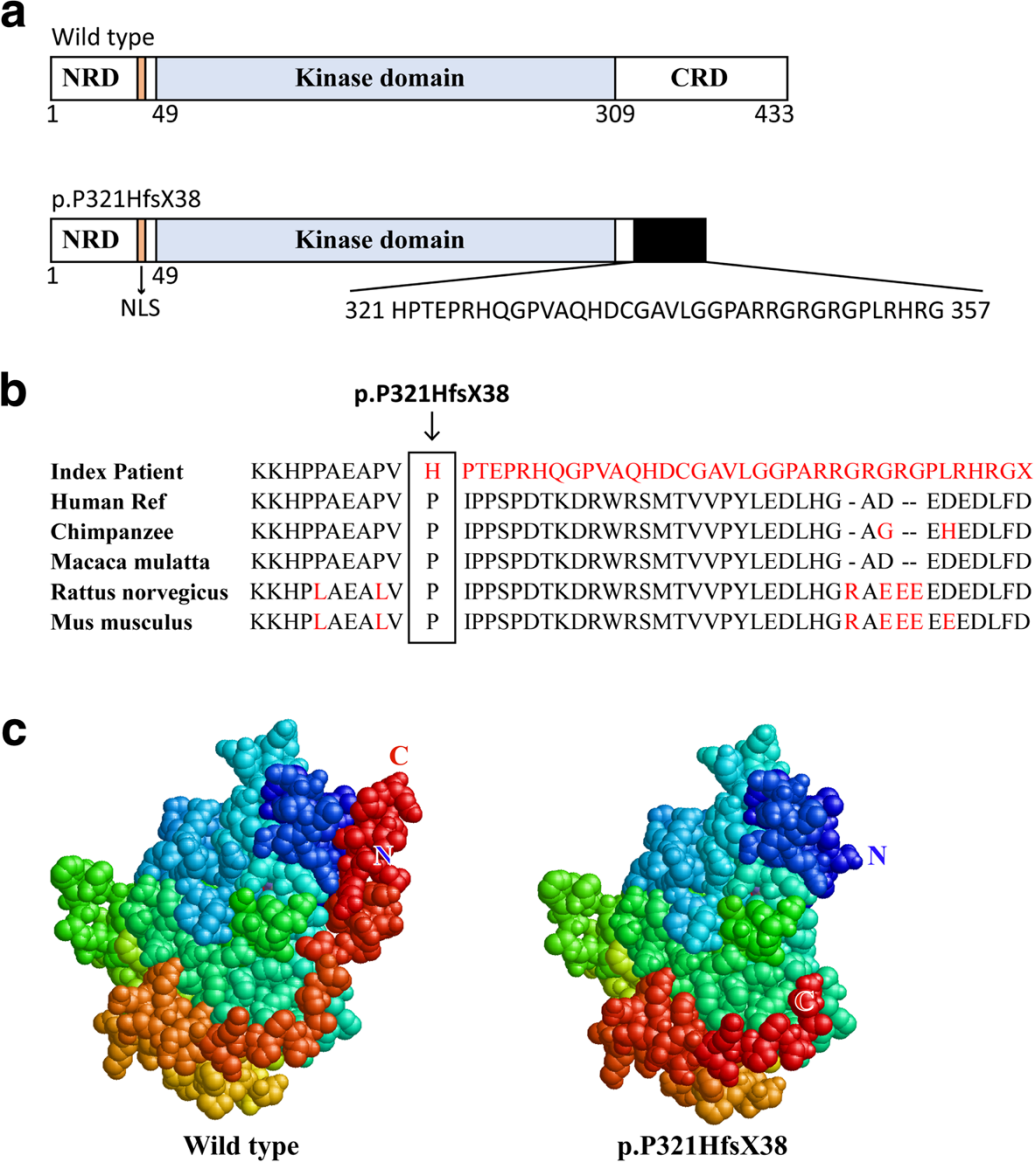
The mutant protein structure. **a** Schematics of the secondary structure or functional domains of the STK11 protein. NLS, Nuclear localization signal, NRD or CRD, N- or C-terminal regulatory domain. **b** Evolutionary conservation of amino acid residues altered by c.962_963delCC (p.P321HfsX38) across different species. **c** The mutant proteins (p.P321HfsX38) were predicted to result in partial loss of the C-terminal domain of the a-helix by Swiss-Model online software compared to the wild type

Through sequence alignment, we found this mutation resulted in a translational frameshift, and the premature stop codon appeared in codon 358 (c.962_963delCC, p.P321HfsX38), which caused a 37-amino-acid-residue change and partial loss of C-terminal regulatory domain (Fig. 2a). Structure prediction demonstrated that the truncated protein lacks part of the C-terminal end (Fig. 2c). What’s more, evolutionary conservation analysis of amino acid residues showed that these impaired amino acid residues were most evolutionary conserved, indicating the mutation was likely pathological (Fig. 2b).

## Discussion and conclusions

PJS is mainly caused by heterozygous germline mutations in *STK11*, and in this study we identified a novel de-novo mutation of *STK11*, c.962_963delCC (p.P321HfsX38), in a Chinese male without a family history. In general, identification of a *STK11* gene mutation in an index patient offers the possibility of a predictive diagnosis in PJS pedigrees, but sometimes an isolated patient not a pedigree of a positive family history is found clinically. In these cases, genetic testing often discovers a germline mutation carried only by the proband but not the parents, which means a de-novo mutation. Though lacking the reminder of a family history, PJS is relatively easy to diagnose according to the obvious mucocutaneous pigmentation and GI hamartomas. In these cases, clinicians should take de-novo mutation into consideration and be aware of the possible cancer risk, so that an appropriate surveillance recommendation can be made.

The STK11 protein is mainly comprised of three major domains: an N-terminal non-catalytic domain, a catalytic kinase domain, and a C-terminal non-catalytic regulatory domain (CRD) [12]. Among the over 400 reported *STK11* mutations detected in patients with PJS or other disorders (HGMD Professional 2016.2) [13], most variants are located in the region of catalytic kinase domain (amino acids 49–309) and result in the absence of kinase activity and disrupting the formation of a complex to maintain kinase activation [14]. Several autophosphorylation sites within this area have been described [15]. As to the CRD of STK11 protein, it is important for binding STRADα/β [16, 17], and mutation within this area could also lessen the AMPKα activity, affect cell polarity,and impair downstream signaling [16]. So this novel mutation broaden the pathological mutation spectrum of the *STK11* gene. It much likely results in the premature termination, which leads to dramatic change and partial loss of CRD, and may contribute to polyp formation and tumorigenesis through various mechanisms, such as loss of growth arrest, apoptosis, and loss of cell polarity.

Although the exact function of STK11 remains largely unknown, studies suggest that it plays a role in cell signaling and apoptosis [18], and several proteins interacting with it (P53, Cdc37, Hsp90, and PTEN) are responsible for PJS phenotype. It is believed that the development of PJS phenotype is due to the elimination of STK11 kinase activity, associating with a loss of growth suppression function [19]. STK11 induces cell cycle arrest and apoptosis on the dependence of P53, and transgenic mice experiments has proven a greater life span reduction and tumor incidence increasing in *STK11* and *P53* both knockout than single gene knockout [20]. Chromatin immunoprecipitation analyses has proven that STK11 is recruited directly to the *P21* gene promoter by P53 and activated *P21* expression [21]. Overexpression of *STK11* could elevate the transcriptional activity of *P53*, while its depletion inhibited P53 functions, which has been observed in patients of PJS-associated GI carcinomas [22, 23]. In our study, computational predictions by software suggested that these mutant proteins lost partial CRD, which possibly impairs the interaction between STK11 and its partners (STRADα/β for example), and eventually cause PJS phenotype, especially the cancerogenesis.

A genotype-phenotype correlation has been sought in PJS, but the relationship between the type and site of *STK11* gene variants and cancer risk is far from clear. Findings supplied by investigators all over the world are difficult to form a complete genotype-phenotype correlation map. PJS-associated cancer should be considered as a complex disease, with gene-gene and gene-environment interactions involved its occurrence and development. The novel mutation detected here also possesses cancer risk, which broadens the spectrum of cancer-related mutation.

Most of the time, PJS is diagnosed when the patient suffers from intestinal obstruction and comes to the surgical emergency, just like the case reported here [24]. With the widely use of endoscopy, PJS patients can be managed intensively to avoid emergency condition and potential cancer risk. People with a positive family history should receive first upper GI endoscopy and colonoscopy at age 8 years, and regular follow-up is necessary [25]. More important, double-balloon enteroscopy (DBE) is the key method to detect and remove small bowel polyps nonoperatively [26–29]. 131 PJS patients who had abdominal surgery for intestinal obstruction before have admitted to our hospital, and with the help of DBE, 113 of them (86.3%) avoid the second open surgery, indicating proper follow-up ensures the patients free from intestinal obstruction and malignancies [30].

In conclusion, we identified a novel heterozygous mutation (c.962_963delCC, p.P321HfsX38) in the *STK11* gene causing PJS in a Chinese male without a PJS family history, and the fact that this patient died of PJS related colon cancer in middle age indicates this mutation a high cancer risk associated one. This study also expands the mutation spectrum of PJS, which forms the fundament of genetic counseling.

## Additional file

Additional file 1: Figure S1. Endoscopic findings of the proband’s only son(III:1). Both gastroscopy (A, B and C) and colonoscopy (D, E and F) discovered no polyp in the digestive tract. A. Gastric fundus. B. Pylorus. C. Duodenal bulb. D. Cecum. E. Hepatic flexure of colon. F. Rectum. (TIFF 915 kb)

## Abbreviations

AMPKα: AMP activated catalytic subunit alpha
GI: Gastrointestinal
PJS: Peutz-Jeghers syndrome
*STK11*: Serine/threonine kinase 11
STRADα/β: STE20 related kinase adaptor alpha/beta

## References

[1] Giardiello FM, Brensinger JD, Tersmette AC, Goodman SN, Petersen GM, Booker SV, Cruz-Correa M, Offerhaus JA (2000). Very high risk of cancer in familial Peutz-Jeghers syndrome. Gastroenterology.

[2] Giardiello FM, Trimbath JD (2006). Peutz-Jeghers syndrome and management recommendations. Clin Gastroenterol Hepatol.

[3] van Lier MG, Wagner A, Mathus-Vliegen EM, Kuipers EJ, Steyerberg EW, van Leerdam ME (2010). High cancer risk in Peutz-Jeghers syndrome: a systematic review and surveillance recommendations. Am J Gastroenterol.

[4] van Lier MG, Westerman AM, Wagner A, Looman CW, Wilson JH, de Rooij FW, Lemmens VE, Kuipers EJ, Mathus-Vliegen EM, van Leerdam ME (2011). High cancer risk and increased mortality in patients with Peutz-Jeghers syndrome. Gut.

[5] Jenne DE, Reimann H, Nezu J, Friedel W, Loff S, Jeschke R, Muller O (1998). Back W, Zimmer M: Peutz-Jeghers syndrome is caused by mutations in a novel serine threonine kinase. Nat Genet.

[6] Hemminki A, Tomlinson I, Markie D, Jarvinen H, Sistonen P, Bjorkqvist AM, Knuutila S, Salovaara R, Bodmer W, Shibata D (1997). Localization of a susceptibility locus for Peutz-Jeghers syndrome to 19p using comparative genomic hybridization and targeted linkage analysis. Nat Genet.

[7] Hearle N, Schumacher V, Menko FH, Olschwang S, Boardman LA, Gille JJ, Keller JJ, Westerman AM, Scott RJ, Lim W (2006). Frequency and spectrum of cancers in the Peutz-Jeghers syndrome. Clin Cancer Res.

[8] Aretz S, Stienen D, Uhlhaas S, Loff S, Back W, Pagenstecher C, DR ML, Graham GE, Mangold E, Santer R (2005). High proportion of large genomic STK11 deletions in Peutz-Jeghers syndrome. Hum Mutat.

[9] Wang ZJ, Churchman M, Avizienyte E, McKeown C, Davies S, Evans DG, Ferguson A, Ellis I, WH X, Yan ZY (1999). Germline mutations of the LKB1 (STK11) gene in Peutz-Jeghers patients. J Med Genet.

[10] Giardiello FM, Welsh SB, Hamilton SR, Offerhaus GJ, Gittelsohn AM, Booker SV, Krush AJ, Yardley JH, Luk GD (1987). Increased risk of cancer in the Peutz-Jeghers syndrome. N Engl J Med.

[11] Biasini M, Bienert S, Waterhouse A, Arnold K, Studer G, Schmidt T, Kiefer F, Gallo Cassarino T, Bertoni M, Bordoli L (2014). SWISS-MODEL: modelling protein tertiary and quaternary structure using evolutionary information. Nucleic Acids Res.

[12] Hanks SK, Quinn AM, Hunter T (1988). The protein kinase family: conserved features and deduced phylogeny of the catalytic domains. Science.

[13] Stenson PD, Mort M, Ball EV, et al. The Human Gene Mutation Database: towards a comprehensive repository of inherited mutation data for medical research, genetic diagnosis and next-generation sequencing studies. Hum Genet 2017;136:665-77. 10.1007/s00439-017-1779-6 PMC5429360 28349240

[14] Boudeau J, Baas AF, Deak M, Morrice NA, Kieloch A, Schutkowski M, Prescott AR, Clevers HC, Alessi DR (2003). MO25alpha/beta interact with STRADalpha/beta enhancing their ability to bind, activate and localize LKB1 in the cytoplasm. EMBO J.

[15] Korsse SE, Peppelenbosch MP, van Veelen W (2013). Targeting LKB1 signaling in cancer. Biochim Biophys Acta.

[16] Forcet C, Etienne-Manneville S, Gaude H, Fournier L, Debilly S, Salmi M, Baas A, Olschwang S, Clevers H, Billaud M (2005). Functional analysis of Peutz-Jeghers mutations reveals that the LKB1 C-terminal region exerts a crucial role in regulating both the AMPK pathway and the cell polarity. Hum Mol Genet.

[17] Baas AF, Kuipers J, van der Wel NN, Batlle E, Koerten HK, Peters PJ, Clevers HC (2004). Complete polarization of single intestinal epithelial cells upon activation of LKB1 by STRAD. Cell.

[18] Shaw RJ, Kosmatka M, Bardeesy N, Hurley RL, Witters LA, DePinho RA, Cantley LC (2004). The tumor suppressor LKB1 kinase directly activates AMP-activated kinase and regulates apoptosis in response to energy stress. Proc Natl Acad Sci U S A.

[19] Mehenni H, Gehrig C, Nezu J, Oku A, Shimane M, Rossier C, Guex N, Blouin JL, Scott HS, Antonarakis SE (1998). Loss of LKB1 kinase activity in Peutz-Jeghers syndrome, and evidence for allelic and locus heterogeneity. Am J Hum Genet.

[20] Wei C, Amos CI, Stephens LC, Campos I, Deng JM, Behringer RR, Rashid A, Frazier ML (2005). Mutation of Lkb1 and p53 genes exert a cooperative effect on tumorigenesis. Cancer Res.

[21] Zeng PY, Berger SL (2006). LKB1 is recruited to the p21/WAF1 promoter by p53 to mediate transcriptional activation. Cancer Res.

[22] Liu L, Du X, Nie J (2011). A novel de novo mutation in LKB1 gene in a Chinese Peutz Jeghers syndrome patient significantly diminished p53 activity. Clin Res Hepatol Gastroenterol.

[23] Korsse SE, Biermann K, Offerhaus GJ, Wagner A, Dekker E, Mathus-Vliegen EM, Kuipers EJ, van Leerdam ME, van Veelen W (2013). Identification of molecular alterations in gastrointestinal carcinomas and dysplastic hamartomas in Peutz-Jeghers syndrome. Carcinogenesis.

[24] van Lier MG, Mathus-Vliegen EM, Wagner A, van Leerdam ME, Kuipers EJ (2011). High cumulative risk of intussusception in patients with Peutz-Jeghers syndrome: time to update surveillance guidelines?. Am J Gastroenterol.

[25] Beggs AD, Latchford AR, Vasen HF, Moslein G, Alonso A, Aretz S, Bertario L, Blanco I, Bulow S, Burn J (2010). Peutz-Jeghers syndrome: a systematic review and recommendations for management. Gut.

[26] Ohmiya N, Taguchi A, Shirai K, Mabuchi N, Arakawa D, Kanazawa H, Ozeki M, Yamada M, Nakamura M, Itoh A (2005). Endoscopic resection of Peutz-Jeghers polyps throughout the small intestine at double-balloon enteroscopy without laparotomy. Gastrointest Endosc.

[27] May A, Nachbar L, Ell C (2005). Double-balloon enteroscopy (push-and-pull enteroscopy) of the small bowel: feasibility and diagnostic and therapeutic yield in patients with suspected small bowel disease. Gastrointest Endosc.

[28] Burke CA, Santisi J, Church J, Levinthal G (2005). The utility of capsule endoscopy small bowel surveillance in patients with polyposis. Am J Gastroenterol.

[29] Parsi MA, Burke CA (2004). Utility of capsule endoscopy in Peutz-Jeghers syndrome. Gastrointest Endosc Clin N Am.

[30] Zhang ZC, Li BR, Li X, Ning SB, Mao GP, Zhang YF, Bu XH, Tang J, M. Z, Jin XW. [Location,growth and clinical outcome of polyps of patients with Peutz-Jeghers syndrome]. Chin J Digestion 2016, 36(9):593.

