# Supplementary figures and images for: Sanger sequencing in exonic regions of STK11 gene uncovers a novel de-novo germline mutation (c.962_963delCC) associated with Peutz-Jeghers syndrome and elevated cancer risk: case report of a Chinese patient
Source: BMC Med Genet. 2017 Nov 15;18:130. doi: 10.1186/s12881-017-0471-y (PMC5688745; doi:10.1186/s12881-017-0471-y)

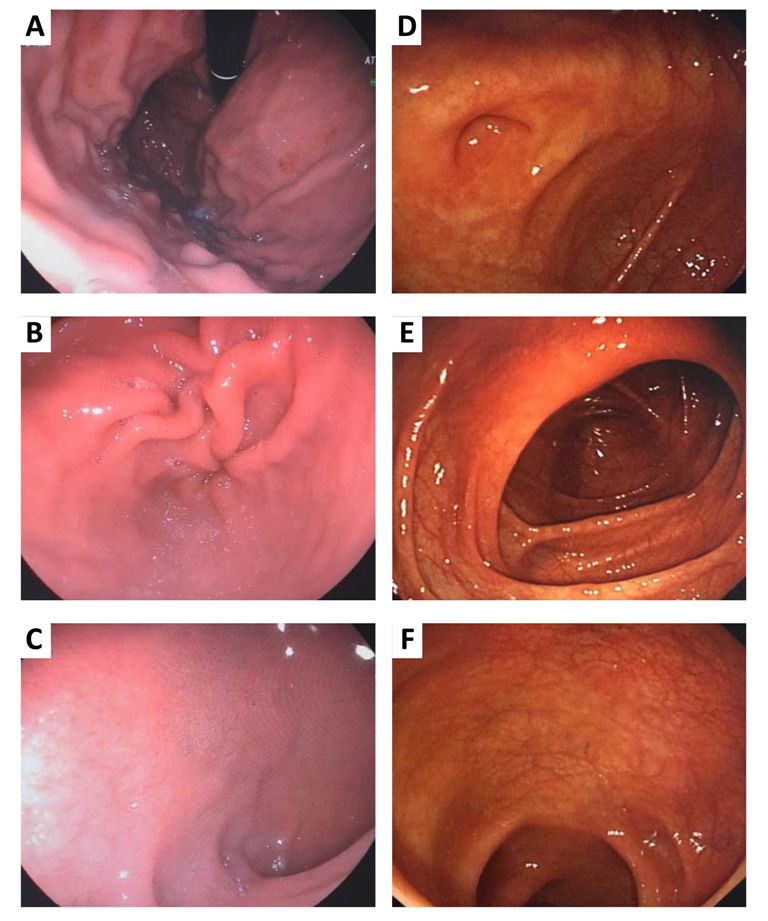

Supplement: Additional file 1: Figure S1. — Endoscopic findings of the proband’s only son(III:1). Both gastroscopy (A, B and C) and colonoscopy (D, E and F) discovered no polyp in the digestive tract. A. Gastric fundus. B. Pylorus. C. Duodenal bulb. D. Cecum. E. Hepatic flexure of colon. F. Rectum. (TIFF 915 kb) [file 12881_2017_471_MOESM1_ESM.tif]
